# *In Vivo* Imaging of α-Synuclein in Mouse Cortex Demonstrates Stable Expression and Differential Subcellular Compartment Mobility

**DOI:** 10.1371/journal.pone.0010589

**Published:** 2010-05-11

**Authors:** Vivek K. Unni, Tamily A. Weissman, Edward Rockenstein, Eliezer Masliah, Pamela J. McLean, Bradley T. Hyman

**Affiliations:** 1 Alzheimer's Research Unit, MassGeneral Institute for Neurodegenerative Disease, MGH Harvard Medical School, Charlestown, Massachusetts, United States of America; 2 Department of Molecular and Cellular Biology and Center for Brain Science, Harvard University, Cambridge, Massachusetts, United States of America; 3 Department of Neurosciences, University of California San Diego, La Jolla, California, United States of America; National Institutes of Health, United States of America

## Abstract

**Background:**

Regulation of α-synuclein levels within cells is thought to play a critical role in Parkinson's Disease (PD) pathogenesis and in other related synucleinopathies. These processes have been studied primarily in reduced preparations, including cell culture. We now develop methods to measure α-synuclein levels in the living mammalian brain to study *in vivo* protein mobility, turnover and degradation with subcellular specificity.

**Methodology/Principal Findings:**

We have developed a system using enhanced Green Fluorescent Protein (GFP)-tagged human α-synuclein (Syn-GFP) transgenic mice and *in vivo* multiphoton imaging to measure α-synuclein levels with subcellular resolution. This new experimental paradigm allows individual Syn-GFP-expressing neurons and presynaptic terminals to be imaged in the living mouse brain over a period of months. We find that Syn-GFP is stably expressed by neurons and presynaptic terminals over this time frame and further find that different presynaptic terminals can express widely differing levels of Syn-GFP. Using the fluorescence recovery after photobleaching (FRAP) technique *in vivo* we provide evidence that at least two pools of Syn-GFP exist in terminals with lower levels of mobility than measured previously. These results demonstrate that multiphoton imaging in Syn-GFP mice is an excellent new strategy for exploring the biology of α-synuclein and related mechanisms of neurodegeneration.

**Conclusions/Significance:**

*In vivo* multiphoton imaging in Syn-GFP transgenic mice demonstrates stable α-synuclein expression and differential subcellular compartment mobility within cortical neurons. This opens new avenues for studying α-synuclein biology in the living brain and testing new therapeutics for PD and related disorders.

## Introduction

Multiple lines of evidence implicate abnormal regulation and aggregation of the synaptic protein α-synuclein in the etiology of Parkinson's Disease (PD) [1]–[2]. Because of this there have been significant efforts to better understand the biology of α-synuclein, including mechanisms relating to its synthesis [3]–[6], degradation [7]–[10], regulation by other proteins [11], and function at synapses [12]–[15]. To date, largely because of technical reasons, these studies have been limited to reduced biochemical preparations, cell culture models and analysis of fixed animal or human tissue. In contrast, the study of other neurodegenerative diseases like Alzheimer's Disease (AD) has recently been advanced by development of *in vivo* multiphoton imaging techniques in mouse models. New insights into the mechanisms of AD involving the formation of extracellular beta-amyloid plaques [16]–[18] and intracellular tau aggregates [19]–[20] have come from these studies that can follow individual plaques and tangles in the mouse brain over time.

The study of PD and other related synucleinopathies would benefit from analogous techniques to study the biology of α-synuclein *in vivo* and its role in neurodegeneration. In this study we detail a new experimental paradigm that allows real-time *in vivo* imaging of fluorescently-tagged human α-synuclein in individual cortical neurons with subcellular resolution over a period of months. We demonstrate that this system is stable and allows for detailed measurements of α-synuclein levels in individual cell bodies and presynaptic terminals. In addition, we use this system to provide the first *in vivo* evidence that α-synuclein protein is differentially mobile within neurons using the fluorescence recovery after photobleaching (FRAP) technique. To date FRAP measurements have been described in numerous systems [21] and to study α-synuclein in other models [22]–[23], but to our knowledge this is the first *in vivo* extension of the technique to mammalian neurons, demonstrating its potential feasibility for studying a wide range of neuronal proteins in living brain.

Our development of these approaches opens lines of inquiry that are difficult to address otherwise. For instance, chronic imaging of individual Syn-GFP expressing cells and presynaptic terminals allows precise analysis of possible changes in these structures over time. In addition, measuring α-synuclein mobility in different subcellular compartments using FRAP can test how its physical state, ability to bind to partners or other geometrical constraints vary within the cell. Understanding these processes in the living brain is of interest since it may lead to new strategies for developing PD therapies.

## Materials and Methods

### Animals

Male Syn-GFP transgenic mice were mated with BDF1 female mice by the MGH Center for Comparative Medicine (CCM). Animals were held in a light-dark cycle, temperature and humidity-controlled animal vivarium and maintained under *ad libitum* food and water diet supplied by the CCM. All experiments were approved by the Subcommittee on Research Animal Care (SRAC) at the MGH and every effort was made to minimize the number of animals used and their suffering.

### Immunohistochemistry

Animals were deeply anesthetized and perfused with a transcardiac approach with ice cold phosphate-buffered saline followed by paraformaldehyde (4%) solution. The brain was quickly removed and placed in paraformaldehyde (4%) at 4 C for a minimum of 24 hr. Next 50–200 µm thick floating sections were cut on a freezing microtome (Microm, HM400). Alpha-synuclein immunohistochemistry was performed after blocking tissue with normal goat serum (10%) at room temperature (RT) for 1 hr. Next sections were stained with a human specific α-synuclein antibody LB509 (Zymed, 1∶100, 4 C for 24 hr) and a Cy3 anti-mouse secondary antibody (Jackson Immunoresearch, 1∶500, RT for 1 hr). All sections were imaged on a Zeiss META laser scanning confocal microscope.

### *In vivo* imaging

The general techniques used for making the “cranial window” have been published previously [24]. After placing the cranial window, isoflurane anesthetized animals were moved to an *in vivo* multiphoton imaging set-up (Olympus Fluoview FV1000 MPE multiphoton microscope microscope with Spectra Physics Mai-Tai tunable laser source, set to 860 nm) and a Z-series stack from a volume of cortex taken (encompassing 10–100 Syn-GFP expressing neurons, >10,000 Syn-GFP expressing presynaptic terminals). Images were analyzed with Image J software (NIH). Somatic Syn-GFP signal from individual neurons was analyzed over time by creating a region of interest (ROI) outlining the somatic compartment for each Syn-GFP expressing cell and measuring the average fluorescence intensity within this ROI. This same ROI was used to measure average fluorescence intensity from the same cell at repeated time points. The average intensity (and standard deviation) from all neurons is plotted as function of time. Presynaptic terminal Syn-GFP expression was analyzed in Image J using the *Analyze Particles* routine (size 0.2–5 µm^2^, default automatic thresholding) to measure terminal density. Histograms of mean terminal density were plotted in Prism 5 (GraphPad). Fluorescence recovery after photobleaching (FRAP) was performed on individual Syn-GFP expressing terminals by first acquiring a baseline image at low laser power (power at sample ∼5–10 mW), then a ROI was drawn around one terminal and this region imaged a high laser power (power at sample ∼45 mW) for 2–5 sec until there was an approximately 75% decrease in terminal signal compared to baseline. Then laser power was returned to the previous low setting and terminals were imaged repeatedly every 1–2 min. Fractional recovery of fluorescence signal was calculated as the fraction of recovered signal over the total bleached signal. FRAP of Syn-GFP expressing cell bodies was done in a similar manner as terminal bleaching except a larger ROI was either made around the whole soma or one half of the soma and a bleaching pulse of ∼5 sec used while moving the focus in the z-direction to bleach the appropriate volume. All animals were maintained at an internal temperature 32–37 C, as measured by rectal probe, with a homeothermic blanket (Harvard Apparatus).

## Results

In this study we perform multiphoton laser scanning microscopy and measure α-synuclein levels in the living brain using a previously described enhanced Green Fluorescent Protein (GFP)-tagged human α-synuclein (Syn-GFP) transgenic mouse line called PDNG78 [25]. This initial characterization demonstrated that fusion of GFP to human α-synuclein's C-terminus and expression under the human Platelet Derived Growth Factor promoter lead to robust expression in a subset of cortical neurons. In addition, this previous work demonstrated increased transgene expression at the mRNA level at ∼3-fold higher levels compared to that found in human brain [25].

**Figure 1:**
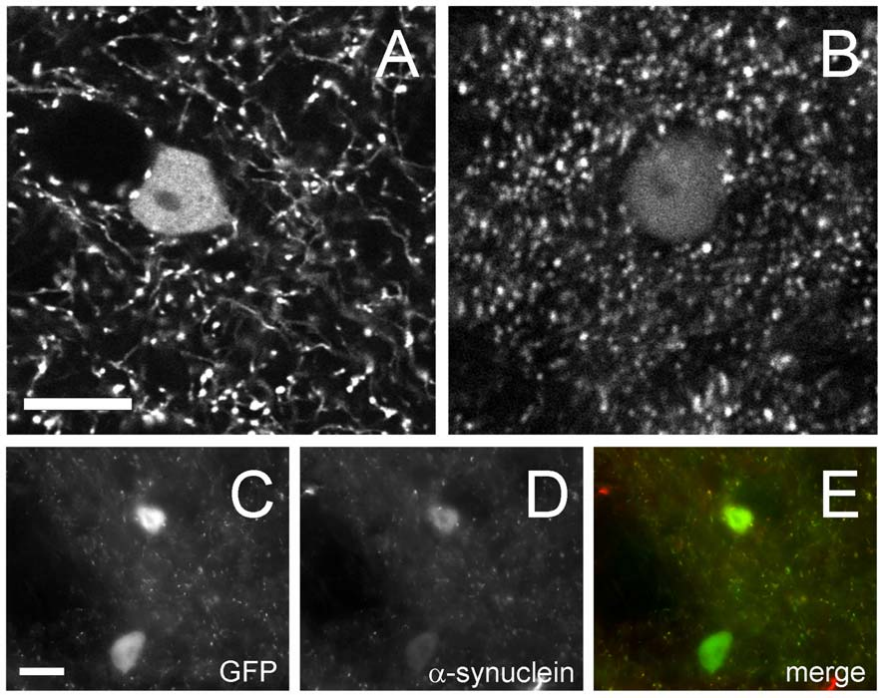
Syn-GFP is present in neuronal cell bodies and presynaptic terminals. A. Fixed tissue confocal image of Syn-GFP in the cortex shows one neuronal soma and that the vast majority of neuropil puncta staining is present within axons. Endogenous GFP fluorescence is shown without antibody labeling. B. *In vivo* multiphoton image of Syn-GFP in the cortex shows staining in one neuronal soma and multiple neuropil puncta. Scale bar for A–B 10 µm. C. Fixed tissue confocal image from the cortex of a Syn-GFP animal showing GFP staining in two somata and in the neuropil. D. Staining in the same section for human α-synuclein. E. Merged image shows colocalization between GFP signal and human α-synuclein, as expected for this fusion protein. Scale bar for C–E 20 µm.

Before starting *in vivo* imaging we used high resolution confocal imaging in fixed cortical tissue to determine the cellular localization of Syn-GFP. This analysis demonstrates that Syn-GFP is detectable in the cell bodies of a sparse subset (∼1–3%) of layer 2/3 cortical neurons and in multiple neuropil puncta (Fig. 1A). Visual inspection shows that the vast majority (>99%) of these puncta are contained within axon-like structures and when paired with previous work showing colocalization of Syn-GFP puncta with the presynaptic terminal marker synaptophysin and electron microscopic localization of Syn-GFP at presynaptic terminals [25] strongly suggests that most neuropil puncta represent presynaptic accumulations of Syn-GFP. Confocal analysis of fixed Syn-GFP tissue also reveals that immunohistochemical staining for human α-synuclein and GFP colocalize as would be expected for this fusion protein (Fig. 1C–E).

Multiphoton imaging demonstrates that a similar pattern of Syn-GFP localization in a sparse subset of neuronal cell bodies and in neuropil puncta can be detected *in vivo* (Fig. 1B) as in fixed tissue. In the *in vivo* case, however, we are unable to visualize individual axons given the lower signal-to-noise ratio of these structures. Another difference between the two techniques is the greater number of neuropil puncta visualized within the plane of focus *in vivo* compared to fixed tissue within a unit area (Fig. 1A–B). This is likely because of the decreased z-axis resolution of *in vivo* multiphoton imaging compared to confocal microscopy.

**Figure 2:**
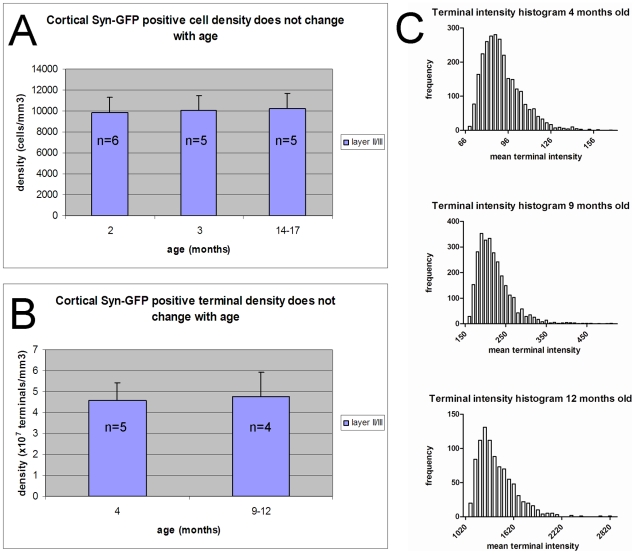
Syn-GFP positive cell body and presynaptic terminal density does not vary with age. A. Group data of average Syn-GFP positive cell body density in layer 2/3 of cortex at different ages demonstrates no difference with age. B. Group data of average Syn-GFP positive presynaptic terminal density in layer 2/3 of cortex at different ages demonstrates no difference with age. C. Representative histograms of mean terminal intensity (in arbitrary units) demonstrate a similar shape at three different ages. n = number of animals, error bars = 1 SD.

**Figure 3:**
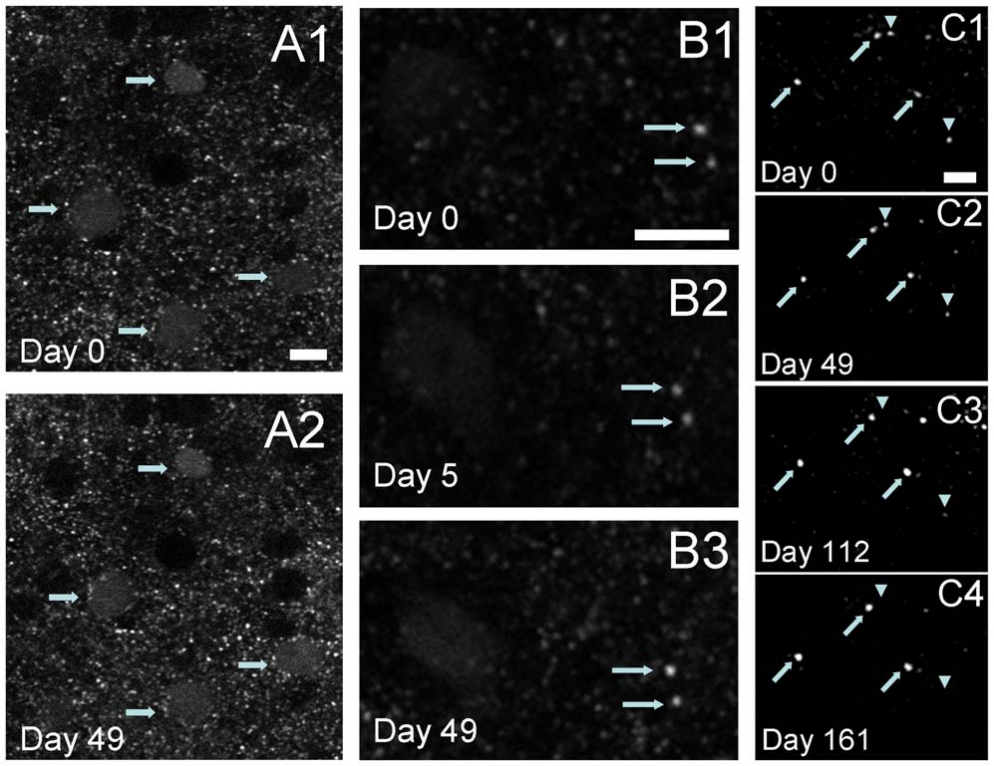
Syn-GFP cell body and high intensity presynaptic terminal expression is relatively stable over time. *In vivo* multiphoton images of the same region at 2 different time points (A1: day 0, A2: day 49) show that Syn-GFP cell body expression is stable over weeks. Arrows show 4 cell Syn-GFP positive bodies that are present at both time points. Scale bar for A1-2 10 µm. *In vivo* multiphoton images of two different regions (B1-3 and C1-4) repeatedly imaged at different time points show that high intensity Syn-GFP terminal expression can be followed over months. Arrows show multiple Syn-GFP positive terminals that are present at all the time points and the arrowheads show high intensity terminals that disappeared over time. Scale bars for B 10 µm and C 7.5 µm.

In order to determine if the density of Syn-GFP positive neurons or presynaptic terminals changes as animals age we measured their respective densities in layer 2/3 of cortex over time. A loss of Syn-GFP positive cells or terminals over time could be a manifestation of α-synuclein-mediated neurodegeneration. In general genetic models of PD have not shown large amounts of frank cell loss [26] but previous studies have not used similar *in vivo* techniques to follow cell or synapse number, which may be more sensitive. In our first cross-sectional study we found that over a period of more than 1 year the density of Syn-GFP expressing neurons (age 2 months: 9830
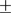
1470 cells/mm^3^ n = 6 animals, age 3 months: 10090
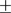
1390 cells/mm^3^ n = 5 animals, age 14–17 months: 10200
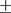
1460 cells/mm^3^ n = 5 animals; Fig. 2A) and presynaptic terminals (age 4 months 4.56
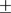
0.87×10^7^/mm^3^ n = 5 animals, age 9–12 months 4.75
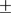
1.16×10^7^/mm^3^ n = 4 animals; Fig. 2B) did not change significantly. In addition, to further characterize the Syn-GFP neuropil puncta we plotted the distribution of mean fluorescence intensity for each punctum within a field of view in an animal 4 months old (Fig. 2C, top panel) and this distribution is positively skewed with a tail towards higher mean puncta intensities. This indicates that Syn-GFP levels can vary by several-fold in different presynaptic terminals. Even with the wide range in Syn-GFP terminal levels detected the general shape of this distribution also did not vary with age (Fig. 2C). Next we performed chronic imaging of the same region of cortex over time. This second longitudinal analysis shows that the pattern of expression of Syn-GFP in particular neuronal cell bodies is stable over a period of weeks (Fig. 3A1-2). A total of 42 individual Syn-GFP positive cell bodies (n = 2 animals) were followed within a total volume of cortex of 4×10^6^ µm^3^ over 49 days and all 42 cells present on day 0 were also present on day 49. In addition, no additional new Syn-GFP positive neurons were detected within this volume. These results suggest that there is no large scale neurodegeneration or synapse loss in Syn-GFP expressing neurons at the ages tested.

The analysis of individual Syn-GFP positive presynaptic terminals over days to months is more complicated than that for Syn-GFP positive cell bodies (described above) since the higher density of terminals makes it difficult to follow the same terminals over the course of days. This is in part because of the small differences in imaging conditions that are inherently present from day to day and the small amount of movement of individual terminals relative to each other that likely occurs. However, we have found that if only the high intensity terminals (those expressing the most Syn-GFP) are selected their density is low enough to follow individual high intensity terminals over time (Fig. 3B1-3, C1-4). This analysis of high intensity terminals showed that individual terminals could be followed over a period of months and that some terminals were stably present while others were lost over time (Fig. 3B1-3, C1-4).

**Figure 4:**
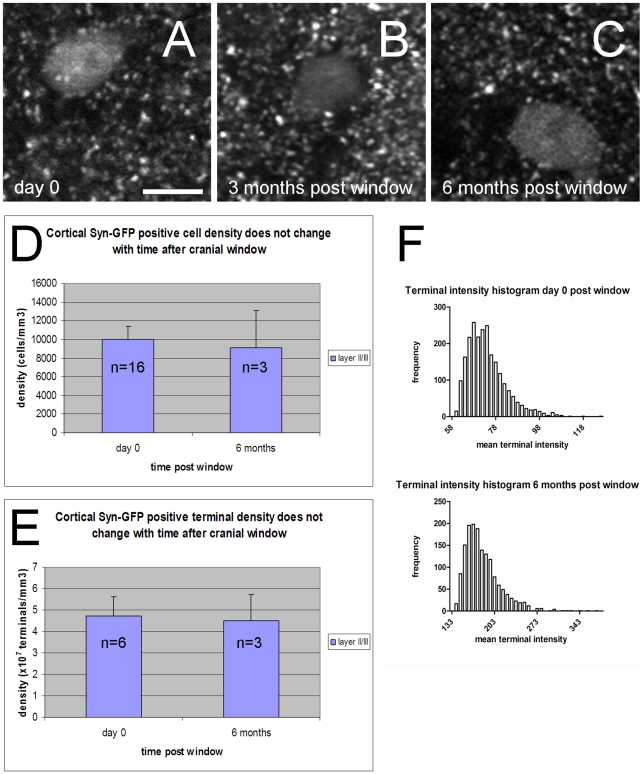
Syn-GFP expression pattern, cell body and terminal density do not vary with time post-window placement. *In vivo* multiphoton images of Syn-GFP in the cortex at 3 different times post-window placement (A: day 0, B: 3 months, C: 6 months) shows a similar pattern of staining. Scale bar 10 µm. D. Group data of average Syn-GFP positive cell body density in layer 2/3 of cortex at different times post window placement demonstrates no significant difference. E. Group data of average Syn-GFP positive presynaptic terminal density in layer 2/3 of cortex at different times post-window placement demonstrates no significant difference. F. Representative histograms of mean terminal intensity (in arbitrary units) demonstrate a similar shape at different times post-window placement. n = number of animals, error bars = 1 SD.

Two different “window” techniques have been developed to perform *in vivo* multiphoton imaging in the cortex of rodents, each with its own advantages and disadvantages [27]–[29]. We tested whether the “glass coverslip” cranial window approach used in this study might produce detectible changes in Syn-GFP over time. Different changes possibly related to the window placement process itself have been suggested in some model systems [18], [30]. In our case, however, repeated imaging of cortical Syn-GFP did not produce any noticeable changes in either the pattern of expression (Fig. 4A–C), density of labeled Syn-GFP cell bodies (day 0 post-window: 10030
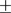
1350 cells/mm^3^ n = 16 animals, 6 months post-window: 9130
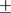
4000 cells/mm^3^ n = 3 animals; Fig. 4D) or presynaptic terminals (day 0 post-window: 4.73
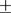
0.88×10^7^/mm^3^ n = 6 animals, 6 months post-window: 4.49
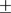
1.26×10^7^/mm^3^ n = 3 animals; Fig. 4E). In addition, the distribution of mean fluorescence intensity for Syn-GFP neuropil puncta maintained the same general shape with time after window placement (Fig. 4F).

Together all the data presented above suggest that this experimental approach provides a powerful new paradigm for visualizing α-synuclein in the living brain, something difficult to do with other methods.

**Figure 5:**
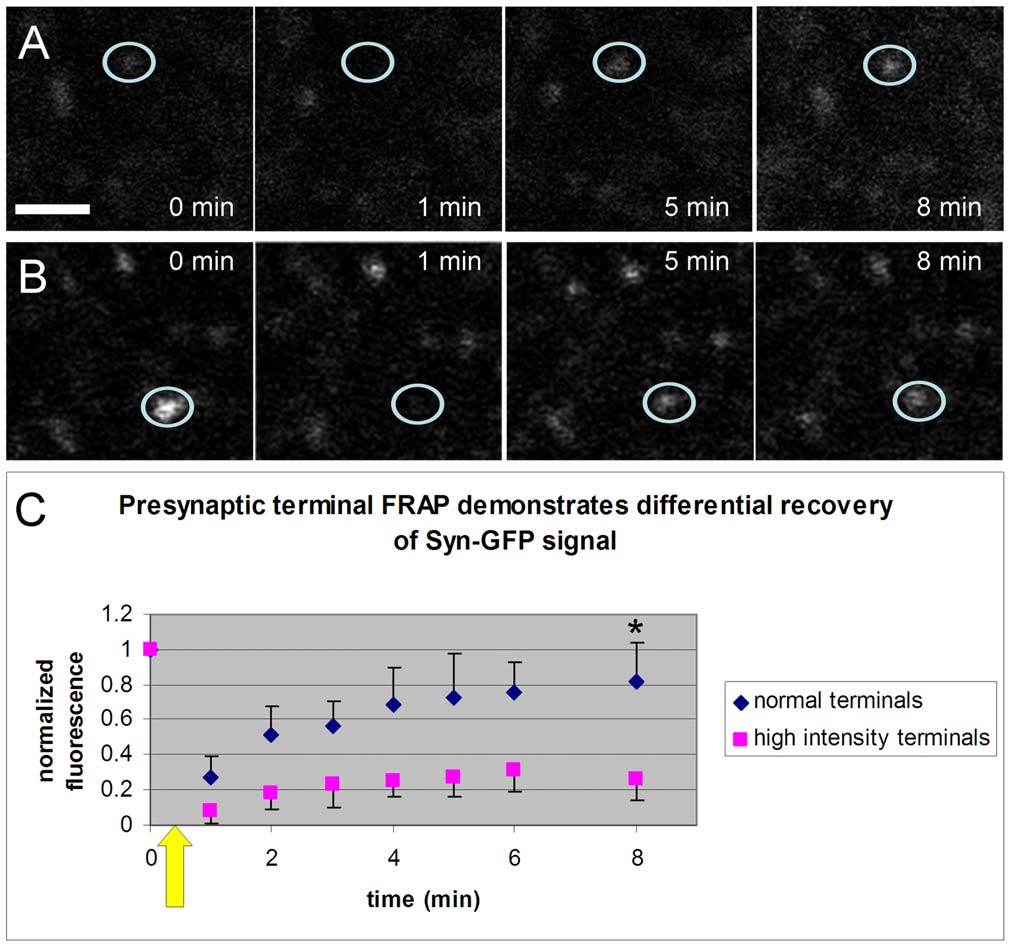
Syn-GFP presynaptic terminal signal recovers differently after photobleaching. A. *In vivo* multiphoton images of the same region of Syn-GFP positive terminals over time. One normal terminal (marked by the white circle) is photobleached just after t = 0. This sequence shows full recovery of Syn-GFP signal over several minutes. B. Similar time sequence as in panel A but in this case one high intensity terminal (marked by the white circle) is photobleached just after t = 0. This sequence shows partial recovery of Syn-GFP signal. Scale bar A and B 5 µm. C. Group data plotting normalized fluorescence before and after presynaptic terminal photobleaching (marked by yellow arrow; normal terminals n = 15 terminals, high intensity terminals n = 10 terminals; n = 5 animals, error bars = 1 SD; * represents statistically significant difference at t = 8 min, t-test p<0.0001).

In order to better characterize the mobility of α-synuclein within neurons in our system and compare this to what has been reported in the literature for hippocampal neurons in culture [22] and *C. elegans* body wall muscle [23], we used the fluorescence recovery after photobleaching (FRAP) technique *in vivo* to photobleach Syn-GFP in individual presynaptic terminals and measure the recovery of this signal over time. Given the positively skewed distribution of mean terminal Syn-GFP intensities we observed (Fig. 2C) and previous work from *C. elegans* suggesting two different populations of inclusions in body wall muscle we decided to selectively photobleach two different populations of presynaptic terminals, high intensity terminals (those in the top decile of the intensity distribution) and “normal” terminals (those outside the top decile). After photobleaching both kinds of terminals we measure a similar rate of recovery of Syn-GFP signal with a t_1/2_∼2 min (Fig. 5) in both cases. This is slower than has been reported in dissociated hippocampal cell cultures for GFP-tagged α-synuclein at presynaptic terminals [22] and in *C. elegans* body wall muscle for yellow fluorescent protein (YFP)-tagged α-synuclein [23]. This suggests a difference in presynaptic terminal α-synuclein mobility in cortical neurons *in vivo* compared to neuronal culture or in worm body wall muscle, the possible causes of which are discussed below. In our experiments fluorescence signal did not recover fully to its pre-photobleaching baseline and the fractional level of recovery was significantly different between high intensity and normal terminals (fractional recovery at 8 min normal intensity terminals: 0.76
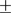
0.22, n = 15 terminals; high intensity terminals: 0.20
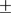
0.12, n = 10 terminals; t-test p<0.0001; n = 5 animals). These fractional levels of recovery are similar to those reported in the two previous studies and can be related to the fraction of immobile α-synuclein species present at terminals, in our case ∼25% in normal terminals and ∼80% in high intensity terminals.

**Figure 6:**
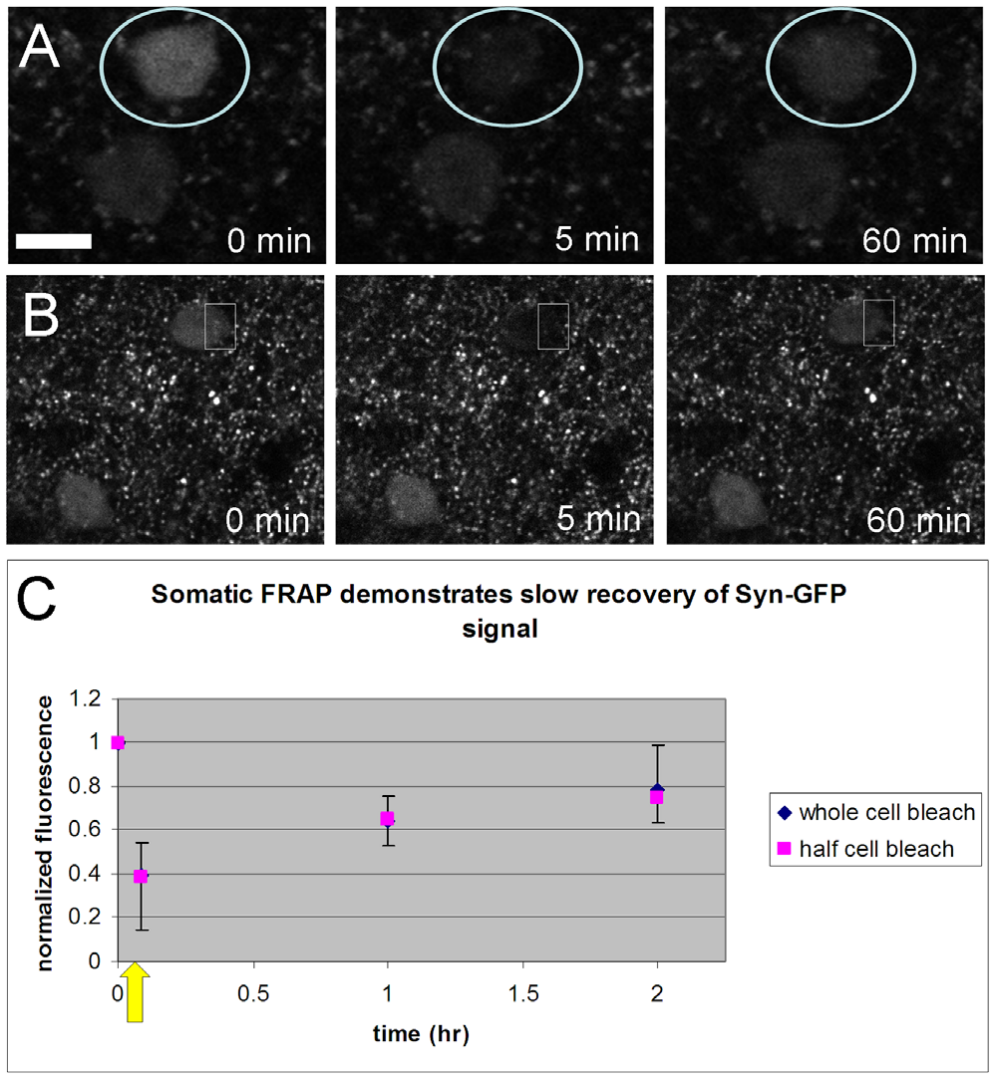
Syn-GFP somatic signal recovers slowly after whole-cell photobleaching and is rapidly mobile during half-cell photobleaching. A. *In vivo* multiphoton images of the same region of Syn-GFP positive cell bodies over time. One soma (marked by the white circle) is photobleached just after t = 0. This sequence shows partial recovery of Syn-GFP signal over 60 min. B. Similar time sequence as in panel A but in this case half the soma (marked by the white rectangle) is photobleached just after t = 0. Scale bar in A 10 µm and in B 20 µm. C. Group data plotting normalized fluorescence before and after photobleaching (marked by yellow arrow; whole-cell bleach: n = 20 cells, half-cell bleach: n = 3 cells; n = 3 animals, error bars = 1 SD).

Next we tested the turnover and mobility of α-synuclein within neuronal cell bodies in our system using the FRAP technique. First we photobleached Syn-GFP throughout the entire cell body and then measured its time course of recovery. The rate of α-synuclein synthesis within cells is an issue of importance since multiple mechanisms controlling this synthesis have been postulated to play a role in PD pathogenesis [3]–[6]. We measure a t_1/2_ of recovery ∼1 hr after photobleaching Syn-GFP throughout the soma (Fig. 6A and C). Given this slower time course the source of Syn-GFP contributing to this recovery is not clear since it may represent the synthesis and maturation of new Syn-GFP molecules, the movement of unbleached Syn-GFP located in other regions (e.g. presynaptic terminals) back to the cell body after the establishment of a concentration difference by somatic FRAP, or a combination of both. In order to better characterize the mobility of α-synuclein within neuronal cell bodies and determine how this movement might play a role in the rate of Syn-GFP recovery after whole-cell bleaching we photobleached Syn-GFP in a region encompassing only one half of the soma and measured its recovery. Somewhat to our surprise, imaging cell bodies after half the soma had been photobleached (bleaching pulse ∼5 sec duration) demonstrated that Syn-GFP within the entire cell body had been greatly reduced to a level equivalent to that seen with whole-cell bleaching (relative signal post whole-cell bleach: 0.39
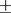
0.15, n = 20 cells; half-cell bleach: 0.38
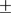
0.24, n = 3 cells; n = 3 animals; Fig. 6B and C). In addition, the t_1/2_ of recovery after half-cell bleaching was essentially identical to that seen with whole-cell bleaching (Fig. 6C). These results strongly suggest that α-synuclein is rapidly mobile within the somatic compartment since essentially all somatic Syn-GFP molecules visit the bleached half of the cell during the 5 sec long bleaching pulse. Given this rapid mobility it is possible that redistribution of Syn-GFP from unbleached regions back to the soma plays a role in the recovery of signal after whole-cell bleaching. Determining the relative contribution of this process (vs. new synthesis and maturation of Syn-GFP molecules) will be an interesting avenue for further study.

## Discussion

Several lines of evidence implicate abnormal α-synuclein regulation and aggregation in the etiology of PD [1]–[2]. Because our understanding of α-synuclein function and dysfunction comes mainly from *in vitro* studies, *in vivo* approaches need to be developed to test implicated mechanisms and potentially reveal new ones that are relevant to PD pathogenesis in the living mammalian brain. The *in vivo* imaging model we have developed measures fluorescently-labeled α-synuclein in individual expressing neuronal cell bodies and presynaptic terminals and can follow them serially over a period of months. It must of course be noted that Syn-GFP may have different biophysical properties than untagged α-synuclein, including an alteration in propensity to aggregate. Recent studies suggest that split fluorescent protein α-synuclein constructs are able to form oligomeric and larger species [31]–[32] but the similarity of these aggregates to those found in PD and other synucleinopathies is not clear. Although more work needs to be done to compare fibrillization kinetics and other biophysical properties of GFP-tagged α-synuclein to the untagged species, the use of *in vivo* imaging in Syn-GFP mice represents a substantial technical advance that can be used to address many questions relating to α-synuclein biology in a relevant context. Our data show that in this transgenic line a sparse subset of cortical neurons expresses Syn-GFP and that the density of expressing neurons does not change substantially with age. In addition, chronic imaging reveals that the subset of Syn-GFP expressing cells seems to be invariant over the course of months, since the rates at which new neurons start to express Syn-GFP or already expressing ones stop expressing the transgene (or degenerate) both appear to be negligible at the ages tested. In addition, the placement of the cranial window itself does not appear to cause detectable changes in Syn-GFP expression. These results suggest that this model system would be particularly useful for experiments that follow α-synuclein expression in individual neurons and synapses over time, both before and after particular manipulations. One possible application for these techniques would be to test the role of different biochemical pathways in determining steady-state α-synuclein levels *in vivo* both in the cell body and at the presynaptic terminal. Pharmacologic or genetic manipulation can first be targeted to specific processes thought to be important for α-synuclein regulation and toxicity such as the ubiquitin-proteasome system, autophagy or α-synuclein phosphorylation. Then Syn-GFP can be measured in individual cells and terminals to test if manipulation causes expression pattern changes or cell loss suggestive of neurodegeneration. This level of sensitivity is difficult to obtain with other techniques and holds the promise of revealing important information about α-synuclein biology in a relevant context.

We have also measured the mobility of α-synuclein at the presynaptic terminal *in vivo*. Interestingly, the t_1/2_ for terminal photobleaching recovery we measure (∼2 min) is slower than that measured in a photobleaching study of GFP-tagged α-synuclein at presynaptic terminals in an acute dissociated hippocampal cell culture system (<10 sec, [22]), or YFP-tagged α-synuclein in *C. elegans* body wall muscle (<10 sec, [23]). In addition to the presynaptic terminal, we have also measured the turnover and mobility of α-synuclein in the somatic compartment of cortical neurons *in vivo*. In contrast to the discrepancy between our presynaptic terminal results and those reported in the literature, the rapid mobility of Syn-GFP we measure within the soma (<5 sec) is in good agreement with these previous studies of α-synuclein mobility. The cause, however, for the difference between our measured presynaptic terminal mobility and the previous studies is not clear but several possibilities exist. One possibility could be that α-synuclein in mouse cortical neurons *in vivo* has the same intrinsic mobility as in hippocampal culture [22] and worm body wall muscle [23] but that differences in the geometry of the axonal tree *in vivo* cause slower measured mobility. In cell culture and in worm body wall muscle nearby reservoirs of unbleached α-synuclein are likely to be much closer to the bleached region than in the mouse cortex where the axonal tree is more complicated and presynaptic terminals (reservoirs of unbleached protein) farther apart. Alternatively, intrinsic mobility may be different in the different systems because the binding affinity of monomeric α-synuclein to lipid membranes in the terminal (a key determinant of mobility) may be higher *in vivo* due to changes in the protein itself (e.g. phosphorylation state), the membrane (lipid composition), or differences in temperature. Another possibility could be the presence of α-synuclein in a multimeric or small aggregate form which would be expected to diffuse less freely. In support of this concept the presynaptic terminal has recently been suggested to be the subcellular location where α-synuclein aggregation begins and microaggregates form [33]. Alpha-synuclein-GFP fusion constructs have been used by many groups to study α-synuclein function in different contexts [22], [34]–[36], but the effect of this construct, if any, on oligomerization in synaptic terminals *in vivo* is unknown. Previous work in this mouse line does demonstrate the presence of granular cytoplasmic aggregates of Syn-GFP in lysosomes [25] but the relationship of these aggregates to those found in synucleinopathies is not clear. Which of these (or other) possibilities give rise to this discrepancy between measured α-synuclein mobility between our data and that reported in the literature is not certain. Our work, however, does suggests specific differences in the mobility of α-synuclein in different subcellular compartments in mouse cortical neurons *in vivo* as compared to other systems that will be interesting to analyze in more detail in the future.

Our measurement of the differential fractional recovery of fluorescence signal after photobleaching in normal terminals (∼75%) and high intensity terminals (∼20%) *in vivo* suggests that the amount of immobile α-synuclein is different in these two groups. Literature values reported for GFP, GFP-tagged wild-type α-synuclein and GFP-tagged α-synuclein bearing the human disease mutation A30P in presynaptic terminals of cultured hippocampal neurons are a fractional recovery of ∼70–80% [22], similar to what we find for normal terminals *in vivo*. In contrast, relatively immobile GFP-tagged synapsin had a lower fractional recovery ∼30–40% [22], which is closer to the fractional recovery we measure for high intensity terminals. In worm body wall muscle two different populations of YFP-tagged α-synuclein, one with a higher level of fractional recovery (∼80%) and another with a lower level (∼40%) have been reported [23]. This result was interpreted as suggesting the presence of two different kinds of inclusions, one with less and one with more immobile α-synuclein protein. We interpret these data similarly, and suggest that there are at least two pools of Syn-GFP in the terminals, one of which has substantially less mobility than the others. Interestingly, however, the high intensity terminal population that has a lower mobility is the same population that shows some evidence for terminal loss over time (Fig 3C).

Our work describes a new model system for studying the biology of α-synuclein in the living brain over a period of months with single cell and even single synapse resolution. The data demonstrate the stability of expression of Syn-GFP within neurons in this transgenic line over time and provide the first *in vivo* measurements of α-synuclein mobility within neurons and terminals. The ability to study α-synuclein with this new level of specificity holds the promise of revealing important insights into the pathobiology of Parkinson's Disease and related synucleinopathies.
